# ISCU interacts with NFU1, and ISCU[4Fe-4S] transfers its Fe-S cluster to NFU1 leading to the production of holo-NFU1

**DOI:** 10.1016/j.jsb.2020.107491

**Published:** 2020-03-06

**Authors:** Kai Cai, Ronnie O. Frederick, John L. Markley

**Affiliations:** Biochemistry Department, University of Wisconsin-Madison, Madison, WI 53706, USA

**Keywords:** Iron-sulfur cluster biogenesis, NFU1, ISCU, Protein-protein interactions, NMR, Small angle X-ray scattering

## Abstract

NFU1 is a late-acting factor in the biogenesis of human mitochondrial iron-sulfur proteins. Mutations in NFU1 are associated with genetic diseases such as multiple mitochondrial dysfunctions syndrome 1 (MMDS1) that involve defects in mitochondrial [4Fe-4S] proteins. We present results from NMR spectroscopy, small angle X-ray scattering, size exclusion chromatography, and isothermal titration calorimetry showing that the structured conformer of human ISCU binds human NFU1. The dissociation constant determined by ITC is *K*_d_ = 1.1 ± 0.2 μM. NMR and SAXS studies led to a structural model for the complex in which the cluster binding region of ISCU interacts with two α-helices in the C-terminal domain of NFU1. *In vitro* experiments demonstrate that ISCU[4Fe-4S] transfers its Fe-S cluster to apo-NFU1, in the absence of a chaperone, leading to the assembly of holo-NFU1. By contrast, the cluster of ISCU[2Fe-2S] remains bound to ISCU in the presence of apo-NFU1.

## 1. Introduction

Fe-S clusters are ubiquitous protein factors that play important roles in a variety of important biological processes including electron transport, central metabolism, gene regulation, DNA repair and replication, and RNA modification (Rouault, 2012; Lill, 2009; Lill and Muhlenhoff, 2006; Johnson et al., 2005; Pain and Dancis, 2016). In most eukaryotic cells, mitochondria constitute the major compartment for Fe-S cluster biosynthesis.

Mitochondrial Fe-S cluster biogenesis is a conserved process that requires the choreographed actions of at least 19 known proteins and can be divided into several steps (Melber and Winge, 2018; Braymer and Lill, 2017; Maio and Rouault, 2015). The initial step is the de novo assembly of Fe-S clusters, which involves the scaffold protein (ISCU), the cysteine desulfurase complex (NFS1-ISD11-ACP, abbreviated as NIA), which catalyzes the conversion of L-cysteine to L-alanine and mobilizes sulfur, a mitochondrial ferredoxin (FDX2 or FDX1), which serves as the electron donor, and frataxin, which may serve as an allosteric factor and/or controls the entries of iron and sulfur (CiofiBaffoni and Nasta, 2017; Pastore and Puccio, 2013; Cai and Markley, 2018; Rouault and Maio, 2017). The second step has been proposed to be release of nascent [2Fe-2S] cluster from the scaffold protein ISCU to mitochondrial monothiol glutaredoxin (GLRX5) facilitated by the mitochondrial chaperone/cochaperone system (Uzarska et al., 2013; Rodriguez-Manzaneque et al., 2002).

The biosynthesis of [4Fe-4S] clusters has been shown to involve a set of proteins including ISCA1, ISCA2, and IBA57 (Beilschmidt et al., 2017; Sheftel et al., 2012; Muhlenhoff et al., 2011). Once synthesized, [4Fe-4S] clusters are further inserted into [4Fe-4S] protein targets such as aconitase, respiratory complex I, and lipoic acid synthase. The insertion of [4Fe-4S] cluster to target proteins involves other ISC proteins such as NFU1, BOLA3, and NUBPL, which likely serve as intermediate [4Fe-4S] carriers and/or late acting factors that are essential for the maturation of specific [4Fe-4S] proteins (Melber et al., 2016; NavarroSastre et al., 2011; Cameron et al., 2011).

A growing number of diseases associated with ISC defects are being discovered through clinical, genetic, and biochemical studies (Rouault, 2012; Rouault, 2015; Stehling et al., 2014; Beilschmidt and Puccio, 2014). Defects in several proteins involved in the biosynthesis and trafficking of [4Fe-4S] clusters are associated with multiple mitochondrial dysfunctions syndromes (MMDS), with NFU1 causing MMDS1, BOLA3 causing MMDS2, IBA57 causing MMDS3, ISCA2 causing MMDS4, and ISCA1 causing MMDS5 (Navarro-Sastre et al., 2011; Cameron et al., 2011; Torraco et al., 2017; Lossos et al., 2015; Shukla et al., 2017; Al-Hassnan et al., 2015; Torraco et al., 2018). These MMDS diseases are characterized by deficiencies in mitochondrial [4Fe-4S] proteins such as lipoic acid synthase (LAS) and those in respiratory complexes I and II.

NFU1, which binds a [4Fe-4S] cluster, was initially thought to be an alternative scaffold protein (Tong et al., 2003); however, NFU1 was later characterized as a late-acting factor required for the maturation of a subset of [4Fe-4S] proteins (Navarro-Sastre et al., 2011; Cameron et al., 2011). NFU1 has been shown to interact with ISCA complexes, and holo-NFU1 has been found to transfer [4Fe-4S] clusters to apoaconitase *in vitro* (Melber et al., 2016; Uzarska et al., 2016; Cai et al., 2016). A similar function has been proposed for the bacterial NFU1 homolog NfuA (McCarthy and Booker, 2017; Bandyopadhyay et al., 2008). An interaction between NFU1 and BOLA3 has been reported, but its appears to be weak and transient (Melber et al., 2016). A recent study failed to detect an interaction between NFU1 and BOLA3 *in vitro* (Nasta et al., 2019). The same study also showed that the [2Fe-2S]GLRX5-BOLA3 complex serves to assemble a [4Fe-4S] cluster on NFU1 in presence of a reducing agent such as DTT (Nasta et al., 2019).

NFU1 consists of two domains: a highly conserved C-terminal domain (CTD) and a less conserved N-terminal domain (NTD). The structures of isolated human CTD and NTD were determined by solution NMR spectroscopy (Cai et al., 2016). Small angle X-ray scattering (SAXS) data indicated that full-length NFU1 adopts a dumbbell-shaped structure, with CTD and NTD connected by a flexible linker region, similar to that of *Arabidopsis thaliana* NFU protein (Cai et al., 2016; Yabe et al., 2008). The conserved CXXC-motif located in the CTD domain on the flexible loop between helix-α2 and strand-β2 provides two Fe-S cluster ligands. Two NFU1 chains are required to supply the four cysteine ligands bound to a [4Fe-4S] cluster. A previous investigation has shown that three of these cluster-linked dimers form a larger assembly in solution (Cai et al., 2016).

In this study, we have used a combination of NMR spectroscopy, small angle X-ray scattering (SAXS), isothermal titration calorimetry (ITC), and size exclusion chromatography (SEC) to detect and explore interactions between human NFU1 and human ISCU. We show that the highly conserved Fe-S cluster binding site and adjacent areas of ISCU interact with the two α-helices of the CTD of NFU1. The dissociation constant was determined to be 1.1 ± 0.2 μM. Although it normally is thought to bind [2Fe-2S] clusters, ISCU has been known for some time to also bind [4Fe-4S] clusters (Unciuleac et al., 2007; Chandramouli et al., 2007; Agar et al., 2000; Colin et al., 2013; Cai et al., 2013; Agar et al., 2000; Smith et al., 2005). In fact, the cluster assembly machinery including cysteine desulfurase has been shown to produce ISCU[4Fe4S] *in vitro* in the presence of high levels of reductant (Chandramouli et al., 2007). This reaction has been discounted by some as a non-physiological artifact (Fox et al., 2015; Webert et al., 2014). However, the results presented here, which show a specific interaction between ISCU and apo-NFU1 along with evidence that ISCU[4Fe-4S], but not ISCU[2Fe-2S], can donate its cluster to apo-NFU1 to generate holoNFU1, support a possible physiological role for ISCU[4Fe-4S].

## 2. Results

### 2.1. NFU1 interacts with the structured conformational form of ISCU

**Fig. 1.**
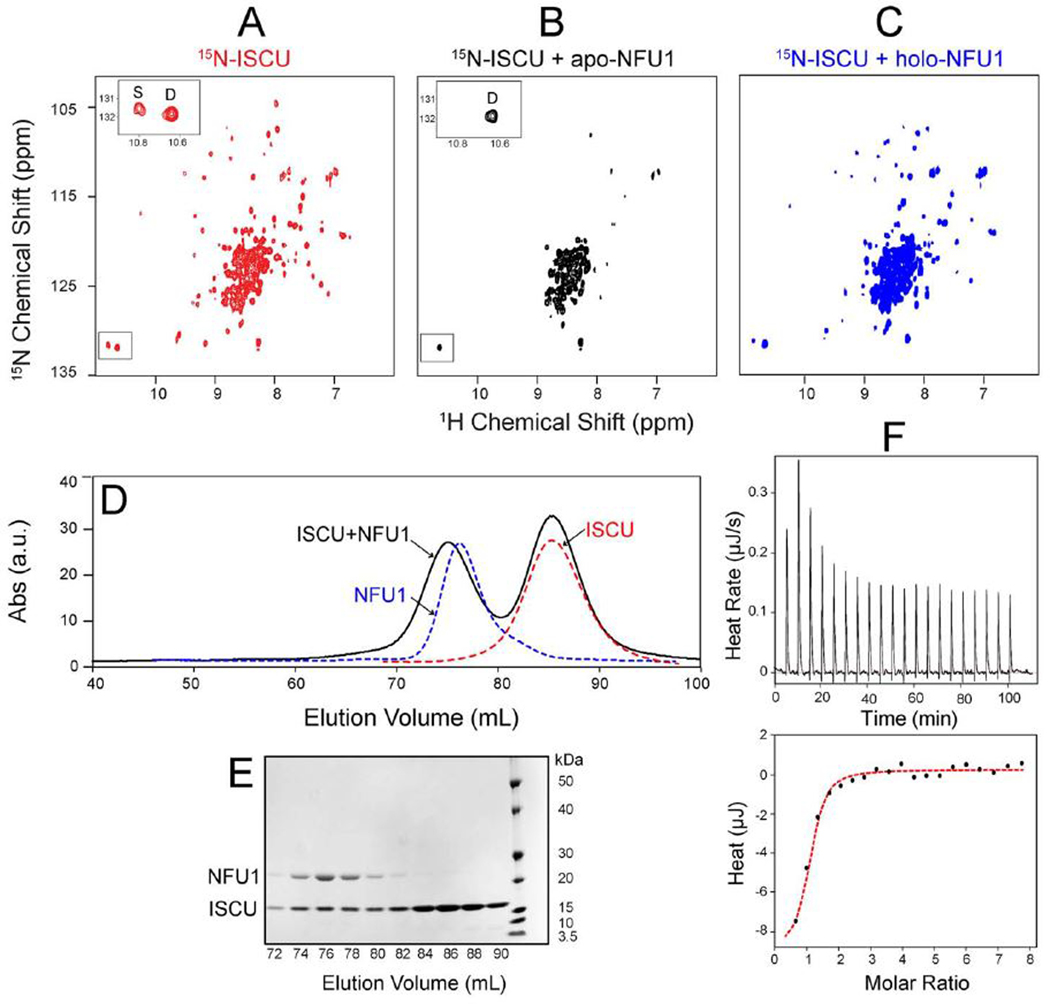
Evidence for a stable interaction between ISCU and apo-NFU1. (*A*) 2D ^1^H,^15^N TROSY-HSQC spectrum of [U-^15^N]-ISCU. *Inset*, expanded view of tryptophan side-chain peaks assigned to the S- and D-states. (*B*) 2D ^1^H,^15^N TROSY-HSQC spectrum of an equimolar mixture of [U-^15^N]-ISCU and unlabeled apo-NFU1. *Inset*, expanded view of tryptophan side-chain peaks. The S-state peak is broadened beyond detection whereas the D-state peak remains visible. (*C*) 2D ^1^H,^15^N TROSYHSQC spectrum an equimolar mixture of [U-^15^N]-ISCU and holo-NFU1. (*D*) Size exclusion chromatography (SEC) profiles of ISCU (red), apo-NFU1 (blue) and a 3:1 molar ratio mixture of ISCU:apo-NFU1 (black). The elution peak with higher molecular weight suggests that apo-NFU1 and ISCU form a stable complex. (*E*) SDSPAGE analysis of the fractions taken from the SEC of 3:1 ISCU:NFU1 (panel *D*) confirms that ISCU and apo-NFU1 form a complex. (*F)* ITC experiment to determine the ISCU-apo-NFU1 binding affinity. Upper panel: peaks indicating heat released after each injection; lower panel: data points fitted to a single 1:1 binding constant to yield thermodynamic parameters (*K*_d_ = 1.1 ± 0.2 μM). (For interpretation of the references to color in this figure legend, the reader is referred to the web version of this article.)

As documented below, NMR spectroscopy, size exclusion chromatography (SEC), and isothermal titration calorimetry (ITC) all provide evidence for interaction between NFU1 and ISCU. Human ISCU in solution has been shown to exist as a ~70:30 mixture of two interconverting conformational states with lifetimes of about one second: a major dynamically disordered form (D) and a minor structured form (S) (Cai et al., 2013). We prepared [U-^15^N]-ISCU by published methods. Its 2D ^1^H,^15^N TROSY-HSQC spectrum contained the expected mixture of poorly dispersed peaks from the D-state and well dispersed peaks from the S-state (Fig. 1A). ISCU has a single tryptophan (W76), which gives rise to two peaks (Fig. 1A, boxed region) assigned individually to the indole ^1^H-^15^N of this residue in the S-state and D-state (Fig. 1A, inset). We produced apo-NFU1 and holo-NFU1 by published procedures (Cai et al., 2016) and prepared equimolar mixtures of [U-^15^N]-ISCU + apoNFU1 and [U-^15^N]-ISCU + holo-NFU1. Comparison of the 2D ^1^H,^15^N TROSY-HSQC spectrum of [U-^15^N]-ISCU + apo-NFU1 (Fig. 1B) with that of [U-^15^N]-ISCU (Fig. 1A) reveals that the well-dispersed signals from the S-form of ISCU (including the W76 indole peak assigned to the S-state) have largely disappeared due to line broadening of peaks beyond detection, as is expected for formation a higher molecular weight complex, whereas the signals from the D-state of ISCU (including the W76 indole peak assigned to the D-state) remained sharp as expected for weak or no interaction (Fig. 1A and B, inset). Similarly, S-state peaks in the 2D ^1^H,^15^N TROSY-HSQC spectrum of [U-^15^N]-ISCU + holo-NFU1 (Fig. 1C) exhibit broadening in comparison to those in the corresponding spectrum of [U-^15^N]-ISCU (Fig. 1C), although the lower effect suggests that holo-NFU1 binds ISCU less tightly than apo-NFU1.

We used SEC analysis to confirm the interaction. The SEC profiles of ISCU (red), apo-NFU1 (blue), and 3:1 mixture of ISCU:apo-NFU1 (black) are shown in Fig. 1D. A peak from the ISCU + apo-NFU1 mixture elutes at a smaller volume (75 mL) than that from apo-NFU1 alone, as expected for a complex. This conclusion is reinforced by SDSPAGE analysis of the SEC fractions (Fig. 1E), which confirms the presence of both ISCU and NFU1 in the peak eluting at 75 mL.

We used ITC to investigate the thermodynamic properties of the interaction between ISCU and NFU1. Titration of ISCU with apo-NFU1 resulted in an exothermic binding reaction that was fitted to a 1:1 binding mode with *K*_d_ = 1.1 ± 0.2 μM (Fig. 1F).

### 2.2. Apo-NFU1 interacts with the region of ISCU that binds Fe-S clusters

**Fig. 2.**
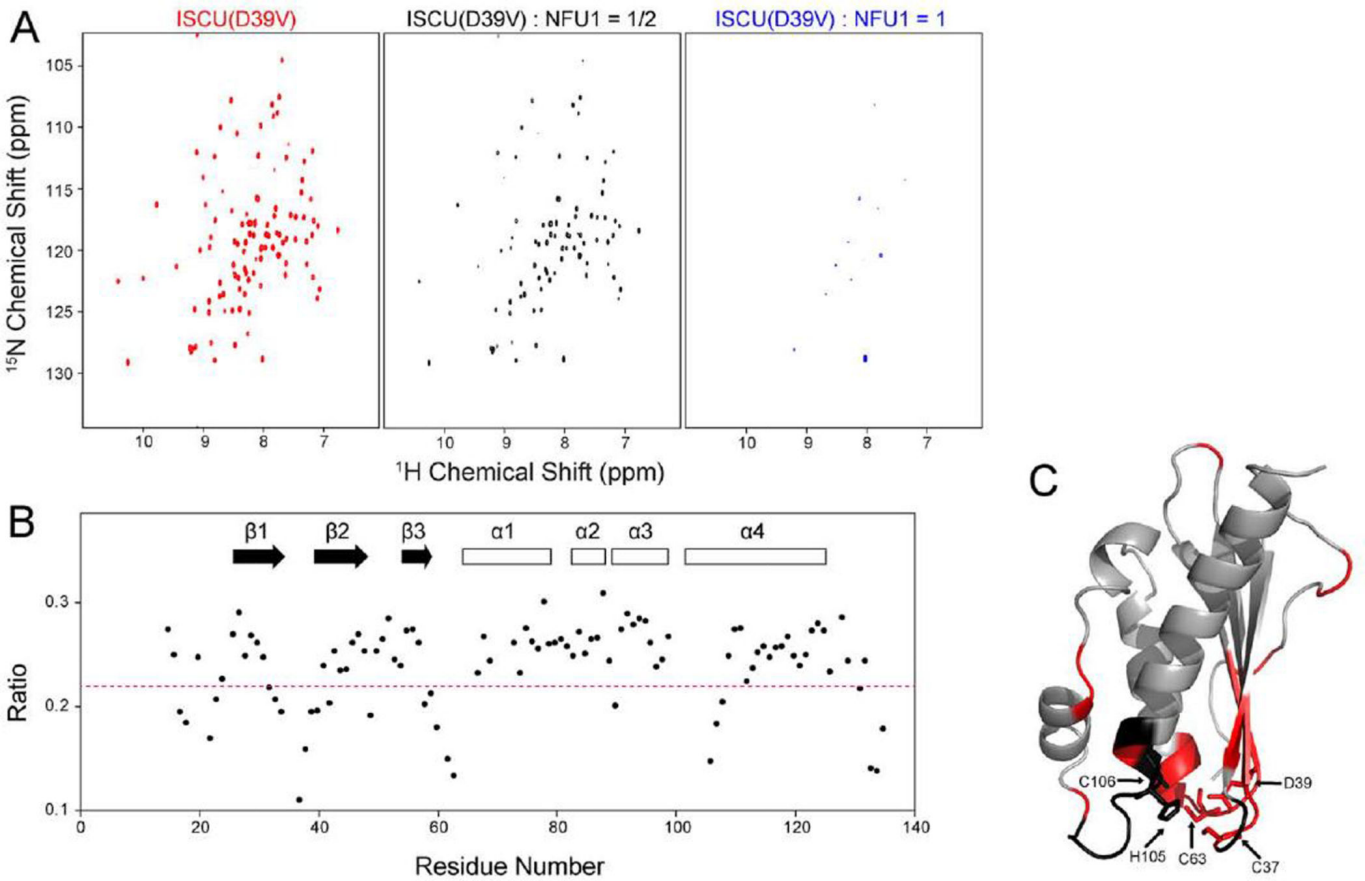
Identification of ISCU residues that bind apo-NFU1. (*A*) 2D ^1^H,^15^N TROSY-HSQC spectra of [U-^15^N]-ISCU(D39V) (left panel), [U-^15^N]-ISCU(D39V) after addition of 0.5 molar equivalent of unlabeled NFU1 (middle panel), and [U-^15^N]-ISCU(D39V) after addition of 1 M equivalent of unlabeled NFU1 (right panel). (*B*) Intensities of assigned peaks in the 2D ^1^H,^15^N TROSY-HSQC spectrum of [U-^15^N]-ISCU(D39V) plus of 0.5 molar equivalent of unlabeled NFU1 divided by those of the spectrum of [U-^15^N]-ISCU(D39V) plotted according to ISCU residue number. (*C*) Color-coded peak intensity ratios from *B* mapped onto the 3D structure of Zn(II)bound mouse ISCU (pdb: 1wfz). Color code: (red) peak intensities significantly decreased (peak ratio < 0.22); (gray) peaks not significantly affected (peak ratio ≥ 0.22); (black) unassigned residue. The Fe-S cluster ligating residues are labeled and represented by sticks. (For interpretation of the references to color in this figure legend, the reader is referred to the web version of this article.)

To avoid the complications from the D-state of ISCU in NMR perturbation mapping studies aimed at identifying ISCU residues that interact with apo-NFU1, we utilized an ISCU variant, ISCU(D39V), which is fully structured and whose NMR peaks have been assigned (bmrb: 27089) (Cai et al., 2018). We collected 2D ^1^H,^15^N TROSY-HSQC spectra of [U-^15^N]-ISCU(D39V) before (Fig. 2A, left panel) and after adding 0.5 (Fig. 2A, middle panel) or 1 (Fig. 2A, right panel) molar equivalent of unlabeled apo-NFU1. As expected from the dissociation constant for the complex, the addition of apo-NFU1 led to progressive disappearance of [U-^15^N]-ISCU(D39V) peaks rather than to separate peaks for free and bound ISCU(D39V). The addition of a sub-stoichiometric amount of unlabeled NFU1 allowed us to track the chemical shift perturbations of the ISCU(D39V) peaks. Given this intermediate exchange regime, we used ratios of the peak intensities in the two spectra (Fig. 2B) to identify residues interacting with apo-NFU1. These peak intensity ratios are color-coded and mapped in Fig. 2C onto the 3D structural model of a closely related ISCU (pdb: 1wfz). The most highly broadened residues (peak ratio < 0.22; colored red) correspond to V17, G18, K22, T23, V32, G33, C37–K42, F58–I67, N90, S107, and M108. Most of these residues map to the region of the Fe-S cluster binding site. The results suggest that the highly conserved Fe-S cluster binding site and its adjacent amino acids form the binding site for NFU1.

### 2.3. The two α-helices on the C-terminal domain of apo-NFU1 interact with ISCU

**Fig. 3.**
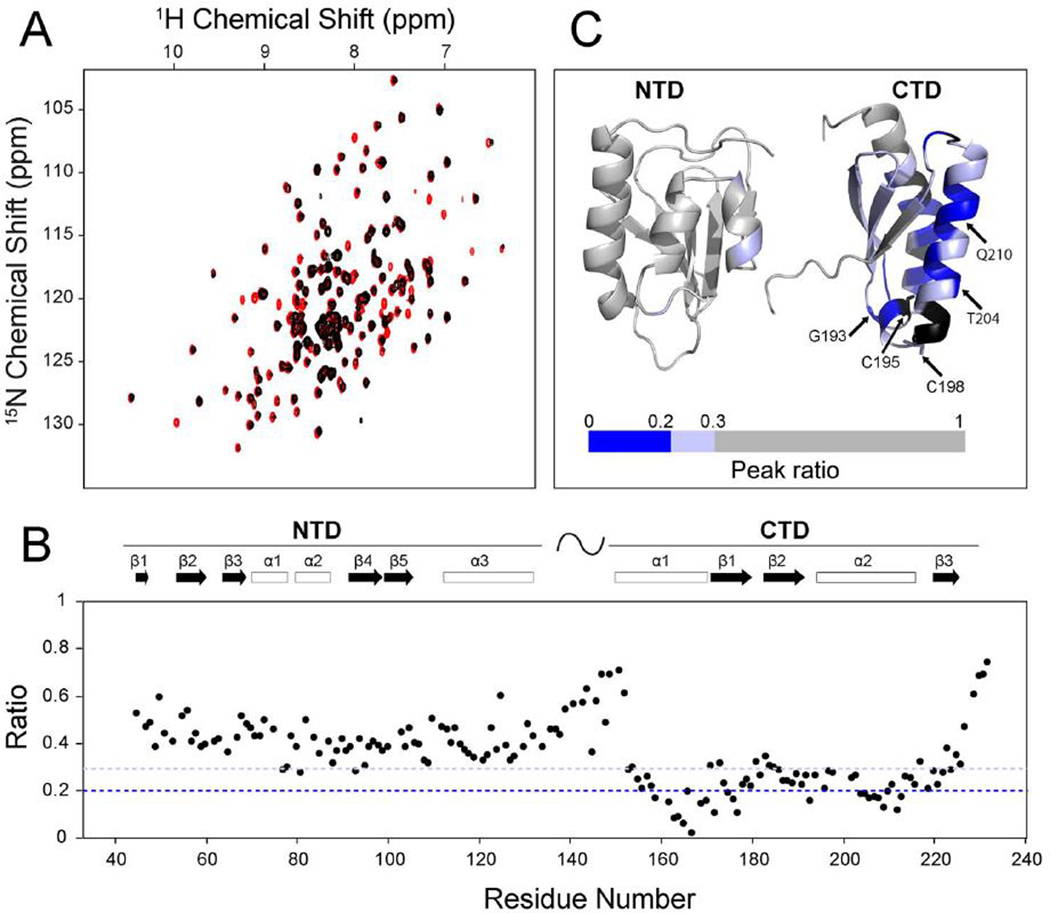
Identification of residues of apo-NFU1 that bind ISCU. (*A*) Overlay of 2D ^1^H,^15^N TROSY-HSQC spectra of [U-^15^N]-NFU1 before (red) and after (black) addition of 0.5 M equivalent of unlabeled ISCU. (*B*) Intensities of assigned peaks in the 2D ^1^H,^15^N TROSY-HSQC spectrum of [U-^15^N]-NFU1 plus of 0.5 M equivalent of unlabeled ISCU divided by those of the spectrum of [U-^15^N]-NFU1 alone plotted according to NFU1 residue number. (*C*) Color-coded peak intensity ratios from *B* mapped onto the 3D structures of the CTD (pdb: 2ltm) and NTD (pdb: 2m5o) domains of NFU1. Color code: (dark blue) peak intensities significantly decreased (peak ratio < 0.2); (light blue) moderate decrease (≥0.2 peak ratio < 0.3); (gray) peaks not significantly affected (peak ratio ≥ 0.3); (black) unassigned residue. (For interpretation of the references to color in this figure legend, the reader is referred to the web version of this article.)

To determine the apo-NFU1 site that binds ISCU, we performed an analogous NMR study with unlabeled ISCU and [U-^15^N]-apo-NFU1. Fig. 3A shows the overlay of 2D ^1^H,^15^N TROSY-HSQC spectra of [U-^15^N]-NFU1 before (red) and after (black) addition of 0.5 M equivalent of unlabeled ISCU. The apo-NFU1 peaks were identified according to assignments deposited in bmrb entry 26801 (Cai et al., 2016). The ratio of peak intensities from apo-NFU1 alone and in the presence of ISCU are plotted as a function of residue number in Fig. 3B. These intensity ratios are color coded and mapped (Fig. 3C) onto the 3D structures of the CTD (pdb: 2m5o) and NTD (pdb: 2ltm) of NFU1 (Cai et al., 2016). Color code: (dark blue) peak intensities significantly decreased, (light blue) moderately decreased (≥0.2 peak ratio < 0.3), (gray) peaks not significantly affected (peak ratio ≥ 0.3), (black) unassigned residue. While most NTD residues of NFU1 were not significantly affected (grey), most CTD peaks fell into the categories of moderate decreased (light blue) or significantly decreased (dark blue) intensity. Residues whose signals were most highly broadened (peak ratio < 0.2, dark blue) are located largely on the two α-helices of CTD. They correspond to NFU1 residues K159, L162–V170, E172, G175–V177, G193, T196, T204–K206, I209, and Q210–L213. The results strongly suggest that the two α-helices on the CTD of NFU1provide a platform for binding ISCU. Taken together, our NMR results indicate that ISCU and NFU1 form a tight complex with the interaction interface formed by the Fe-S cluster binding site of ISCU and the two α-helices on the CTD of NFU1, which are in close proximity to the two conserved cysteine residues (C195 and C198) that bind the [4Fe-4S] cluster in holo-NFU1.

### 2.4. Structural model of the ISCU:apo-NFU1 complex

**Fig. 4.**
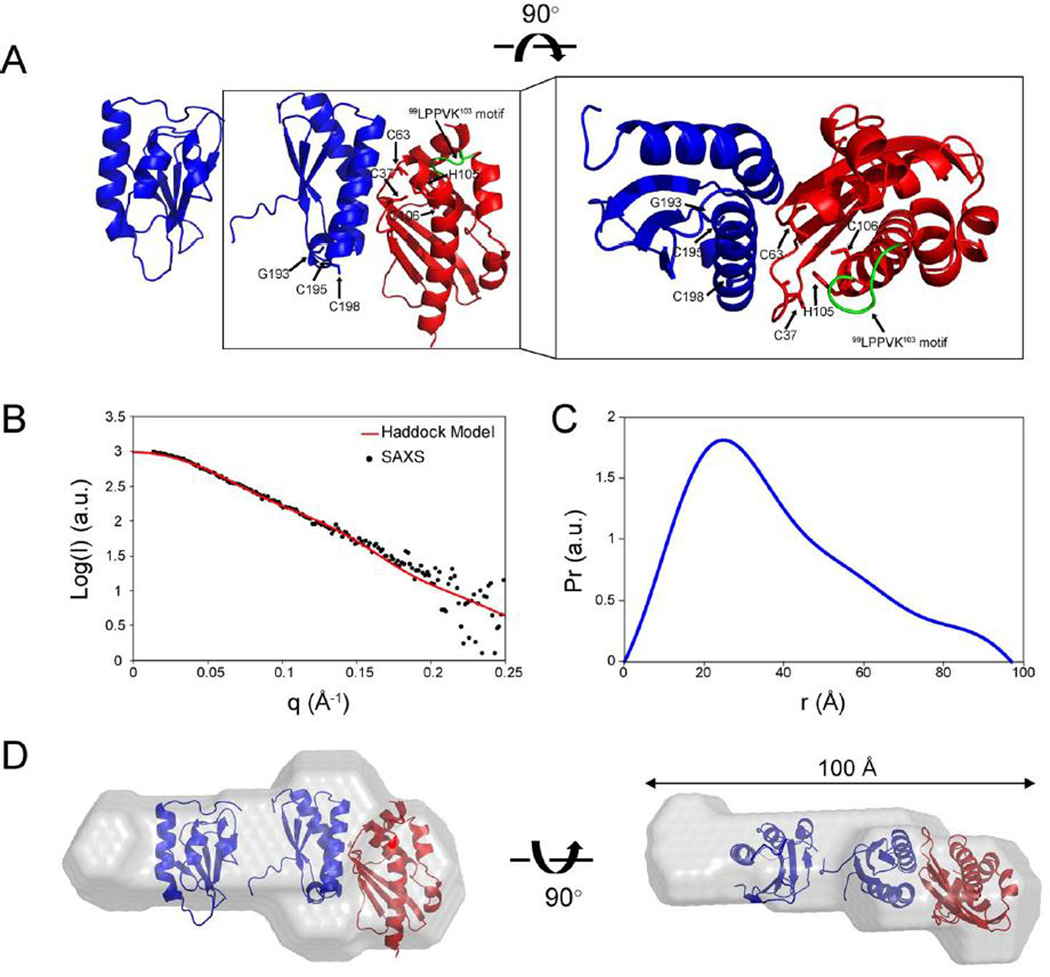
HADDOCK model of the NFU1:ISCU complex and its validation by SAXS. (*A*) Structural model of the complex between apo-NFU1 and ISCU generated by the HADDOCK2.2 webserver (van Zundert et al., 2016). (*B*) Experimental SAXS data from the apo-NFU1:ISCU complex (black dots) overlaid with theoretical scattering curves computed from the HADDOCK structural model (*χ*^2^ = 1.36). (*C*) Pairwise distribution function (*P*_r_) plot derived from the experimental SAXS data. (*D*) Superposition of the HADDOCK structural model of apo-NFU1:ISCU on the *ab initio* dummy-atom envelope calculated from the SAXS data for apo-NFU1:ISCU.

We utilized the structural models of ISCU and the CTD of apo-NFU along with ambiguous structural restraints derived from the NMR peak perturbations as input to HADDOCK software (van Zundert et al., 2016). The 200 calculated structures were clustered, and the topscoring cluster (consisting of 111 structures) had a HADDOCK score of −114.3 ± 4.6 and RMSD 1.3 ± 0.7 (Table 2). In the highest scoring HADDOCK structural model (Fig. 4A), the Fe-S cluster binding site of ISCU interacts with the two α-helices on the CTD of NFU1, and the αhelices contact the two cysteine-containing loops of ISCU, with ^34^APACGD^39^ next to helix-α2 and ^61^FGCGS^65^ next to helix-α1.

To validate the structural model, we carried out small angle X-ray scattering (SAXS) on a solution containing apo-NFU1:ISCU freshly isolated by SEC (as shown in Fig. 1D). The SAXS profile yielded *R*_g_ = 35.8 Å by Guinier approximation. The maximum end-to-end distance for the complex derived from the pairwise distribution function (*P*_r_) plot was *D*_max_ = 97.6 Å (Fig. 4 A–B and Table 1). The molecular mass of apo-NFU1:ISCU as determined by the *V*c approach (Rambo and Tainer, 2013) was 42.8 kDa, consistent with the theoretical value of 41.7 kDa for a 1:1 complex (Table 1). Comparison of experimental SAXS data and the theoretical scattering curve using CRYSOL software (Svergun et al., 1995) yielded a *χ*^2^ value of 1.36 (Fig. 4B), indicating good agreement between the two. The shape envelope of the *ab initio* model of apo-NFU1:ISCU (gray) generated by the DAMMIF program superimposed well on the structural model generated by HADDOK modeling (Fig. 4D). The empty space in the SAXS envelope is attributed to the N-terminal amino acid residues of apo-NFU1 that were present in the sample used for the SAXS experiment but not modeled in the NMR structure of the NTD of NFU1 (pdb: 2ltm) used in creating the HADDOCK model.

### 2.5. ISCU[4Fe-4S] transfers its cluster to apo-NFU1, but ISCU[2Fe-2S] does not

**Fig. 5.**
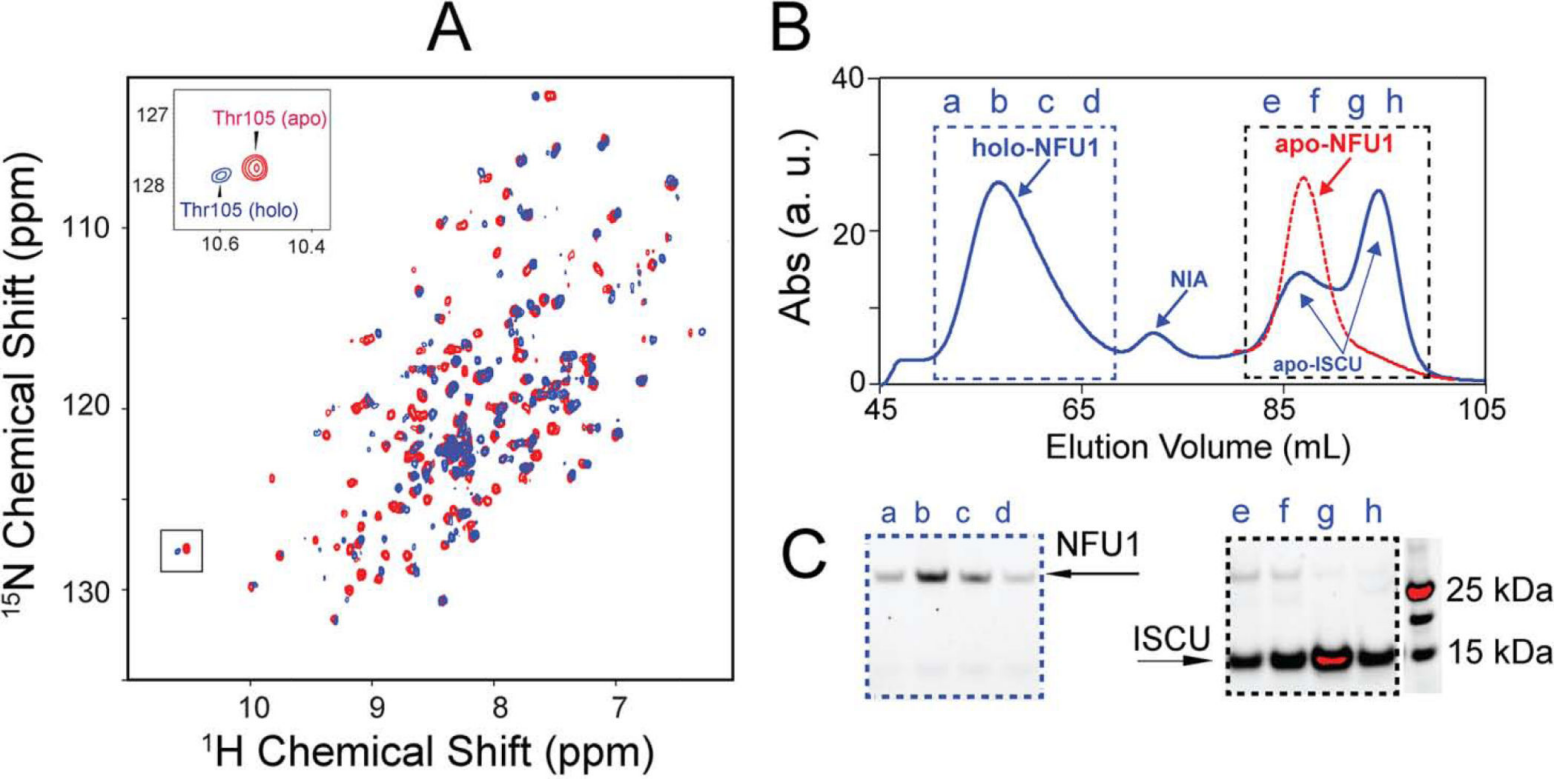
Evidence that ISCU[4Fe-4S] transfers its Fe-S cluster to apo-NFU1. (*A*) Overlay of 2D ^1^H-^15^N TROSY-HSQC spectra of apo-[U-^15^N]-NFU1 (red) and [U-^15^N]NFU1 following the addition of two molar equivalents of ISCU[4Fe-4S] (blue). *Inset*, ^1^H-^15^N peaks corresponding to Thr105 in apo- and holo-NFU1. (*B*) SEC profile of apo-NFU1 (red dashed line) and of the product resulting from the addition of ISCU[4Fe-4S] to apo-NFU1 (blue). (*C*) SDS-PAGE of the SEC elution fractions denoted in panel B. (For interpretation of the references to color in this figure legend, the reader is referred to the web version of this article.)

As noted above, several studies have shown that ISCU can assemble both [2Fe-2S] and [4Fe-4S] clusters (Unciuleac et al., 2007; Chandramouli et al., 2007; Agar et al., 2000; Colin et al., 2013). We prepared ISCU[4Fe-4S] in vitro in an anaerobic chamber by using the NFS1-ISD11-Acp complex (NIA) as the cysteine desulfurase and excess DTT as the reducing agent. The UV–vis spectrum of the reaction mix after reconstitution demonstrated a single broad peak at ~ 410 nm, characteristic of a [4Fe-4S] cluster (Supplementary Fig. 1, red line). The [4Fe-4S] cluster yield was ~ 70% calculated by using the extinction coefficient ε_410nm_ = 15,000 M^−1^ cm^−1^ for a [4Fe-4S]^2+^. In the anaerobic chamber, following dialysis of the ISCU[4Fe-4S] product to remove free iron and sulfide, we added a solution of [U-^15^N]-apo-NFU1. Following a 30-min incubation at room temperature, we transferred the mixture to a 5 mm NMR tube with a gas-tight seal. Fig. 5A is an overlay of the 2D ^1^H,^15^N TROSY-HSQC spectrum of the reaction mixture (plotted in blue) with the spectrum of the starting material [U-^15^N]-apoNFU1 (plotted in red). The spectrum in blue corresponds closely to the published spectrum of holo-NFU1, and the spectrum in red matches the published spectrum of apo-NFU1 (Cai et al., 2016). The comparison (Fig. 5A, boxed region and inset) reproduces the peaks assigned to Thr105 in apo- and holo-NFU1 (Cai et al., 2016). The lack of a blue peak overlaying the red peak, indicates that most of the apo-NFU1 has been transformed to NFU1[4Fe-4S].

We showed previously that NFU1[4Fe-4S] forms a hexamer (Cai et al., 2016). To test whether the product of adding ISCU[4Fe-4S] to apo-NFU1 also forms a hexamer, an aliquot of the sample used for NMR spectroscopy was subjected to anaerobic SEC analysis. The SEC profile showed three major elution peaks with elution volumes of 60 mL, 87 mL, and 98 mL, whose identities were determined by SDS-PAGE (Fig. 5B–C). The peak that eluted at 60 mL (Fig. 5B, blue box), which contained only NFU1 by SDS-PAGE (Fig. 5C, blue box, the four bands a–d correspond to four SEC fractions a–d in Fig. 5B), had the same elution volume as the 160 kDa hexameric holo-NFU1 investigated previously (Cai et al., 2016). UV–vis spectrum of fraction a–d pooled together showed a single broad peak at ~ 410 nm, indicating the presence of a [4Fe-4S] cluster (Supplementary Fig. 2, red). By contrast, the elution volume of apo-NFU1 is about 88 mL (red dashed line). The peak that eluted at 87 mL contained predominantly ISCU and a slight amount of NFU1, while the peak at 98 mL contained only ISCU (Fig. 5BC). The molecular weights of these two ISCU species (30 kDa and 15 kDa) correspond, respectively, to those of dimeric and monomeric ISCU. Both dimeric and monomeric ISCU were in apo form as the UV–vis spectrum of fraction e–h pooled together showed no peaks pertaining to Fe-S clusters (Supplementary Fig. 2, black). These results demonstrate that ISCU[4Fe-4S] transfers its [4Fe-4S] cluster to apo-NFU1, leading to formation of hexameric holo-NFU1.

**Fig. 6.**
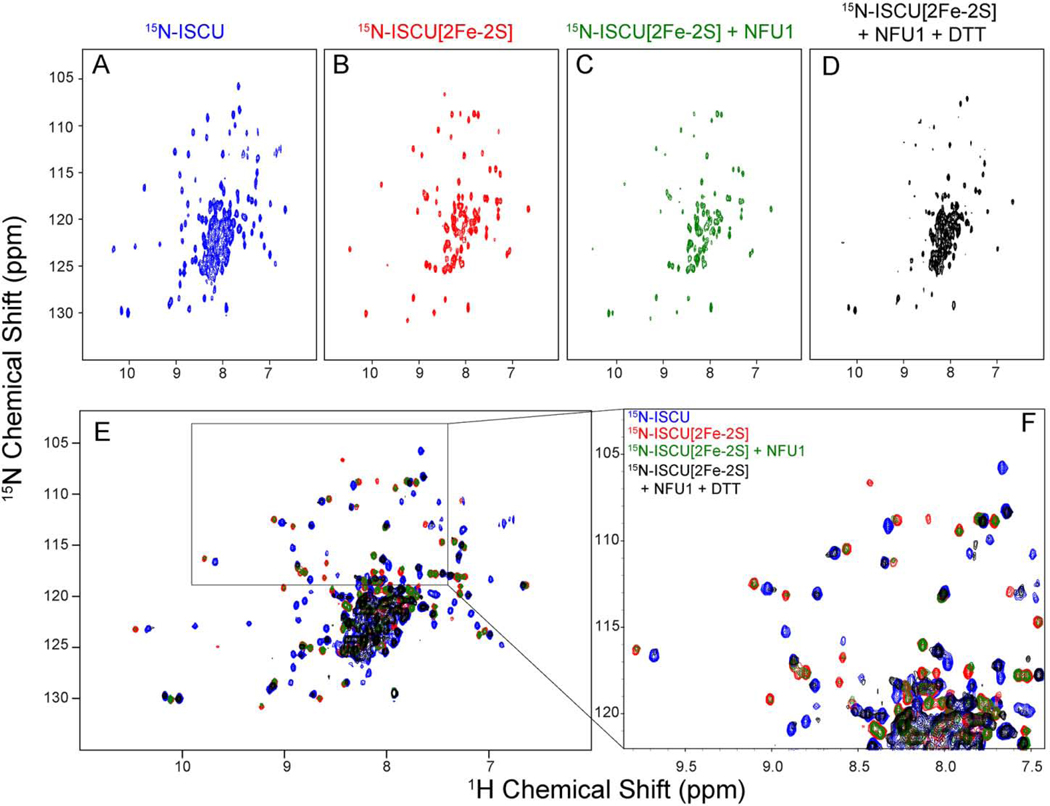
Evidence that ISCU[2Fe-2S] does not transfer its Fe-S cluster to apo-NFU1. 2D ^1^H,^15^N TROSY-HSQC spectra of (*A*) [U-^15^N]-ISCU, (*B*) [U-^15^N]-ISCU[2Fe-2S], and (C) [U-^15^N]-ISCU[2Fe-2S] following the addition of 0.5 molar equivalent of apo-NFU1. (*D*) [U-^15^N]-ISCU[2Fe-2S] following the addition of 0.5 molar equivalent of apo-NFU1 and 5 mM DTT. (*E*) Overlaid spectra from panels *A* ([U-^15^N]-ISCU, blue), *B* ([U-^15^N]-ISCU[2Fe-2S], red), and C ([U-^15^N]-ISCU[2Fe-2S] + NFU1, green). (*F*) Expansion of a region of *D*. (For interpretation of the references to color in this figure legend, the reader is referred to the web version of this article.)

Because ISCU assembles both [2Fe-2S] and [4Fe-4S] clusters, it was of interest to investigate whether the [2Fe-2S] cluster from ISCU[2Fe2S] is transferred similarly to apo-NFU1. We used a published method to prepare and purified [U-^15^N]-ISCU[2Fe-2S] in the anaerobic chamber (Cai et al., 2017). One aliquot was transferred to an NMR tube with a gas-tight seal, and a second aliquot of [U-^15^N]-ISCU[2Fe-2S] was mixed with 0.5 molar equivalent of apo-NFU1. The [U-^15^N]-ISCU[2Fe2S] + apo-NFU1 mixture was incubated in the anaerobic chamber for 10 h before it was transferred to an NMR tube with a gas-tight seal. Fig. 6 compares the 2D ^1^H,^15^N TROSY-HSQC spectra of (*A*) [U-^15^N]ISCU, (*B*) [U-^15^N]-ISCU[2Fe-2S], and (*C*) the 2:1 mixture of [U-^15^N]ISCU[2Fe-2S] and apo-NFU1. Whereas the spectrum of labeled ISCU exhibits ^1^H-^15^N peaks for the D- and S-states, signals from the D-state are missing from the spectrum of [U-^15^N]-ISCU[2Fe-2S]. This indicates, as expected, that cluster binding stabilizes the S-state. Cluster binding leads to shifting of some peaks and the disappearance of some peaks from residues near the paramagnetic Fe-S cluster, which is in the oxidized state. The addition of sub-stoichiometric apo-NFU1 led to broadening of peaks from [U-^15^N]-ISCU[2Fe-2S] suggesting that the two proteins interact. The spectra are overlaid in Fig. 6E with [U-^15^N]ISCU in blue, [U-^15^N]-ISCU[2Fe-2S] in red, and [U-^15^N]-ISCU[2Fe2S] + apo-NFU1 in green. If cluster transfer from [U-^15^N]-ISCU[2Fe2S] to apo-NFU1 had occurred, green signals should overlay the blue signals from [U-^15^N]-ISCU; if not, green signals should overlay with red signals from [U-^15^N]-ISCU[2Fe-2S]. Because the latter is observed (better shown in the expansion in Fig. 6F), we conclude that, although the proteins interact, cluster transfer has not occurred.

It is known that the addition of a reducing agent to [2Fe-2S]ISCU leads to the generation of [4Fe-4S] clusters. When we added DTT at a concentration of 5 mM to the mixture of [U-^15^N]-ISCU[2Fe-2S] and apo-NFU1, the resulting spectrum shown in black (Fig. 6D) corresponded to that of [U-^15^N]-apo-ISCU shown in blue (Fig. 6E,F), suggesting that [2Fe-2S]ISCU can assemble a [4Fe-4S] cluster on NFU1. However, this experiment does not answer the question whether the reduction of two [2Fe-2S] clusters to one [4Fe-4S] cluster occurs before, after, or at the same time as cluster transfer. The broadening of and lower resolution of Fig. 6D compared to Fig. 6A is the result of the relatively weak interaction between holo-NFU1 and ISCU as described above (Fig. 1C).

## 3. Discussion

Extensive investigations over the past two decades have identified many new components and established key steps in the mitochondrial machinery involved in the biosynthesis of Fe-S proteins. Nevertheless, the roles and molecular mechanisms of many proteins implicated in this machinery are yet to be established. [4Fe-4S] clusters are the most abundant type of cluster in mitochondria, and the mechanisms by which they are inserted are the focus of ongoing investigations. It has been shown that the generation of [4Fe-4S] clusters from [2Fe-2S] clusters *in vivo* requires a set of proteins including ISCA1, ISCA2, and IBA57 (Sheftel et al., 2012; Muhlenhoff et al., 2011; Brancaccio et al., 2014). NFU1 and BOLA3 function together in [4Fe-4S] cluster transfer from the ISCA complex to apo-client proteins (Melber et al., 2016).

Here, we report experimental results from NMR, ITC, SEC, and SAXS that provide novel evidence that human ISCU, a scaffold for Fe-S cluster assembly, and NFU1, a protein involved in maturation of proteins that contain [4Fe-4S] clusters, form a specific complex in which amino acid residues of ISCU known to serve as Fe-S cluster ligands bind to two helices of NFU1 adjacent to its cluster binding cysteine residues. Moreover, we show that when NFU1 and ISCU[4Fe-4S] are mixed, the cluster is transferred from ISCU to NFU1 leading to the formation of holo-NFU1. When NFU1 and ISCU[2Fe-2S] are mixed under similar conditions; however, no cluster transfer is observed. Together, the results suggest that ISCU[4Fe-4S] may play an important physiological role in an alternative pathway from that described in the previous paragraph for the maturation of apo-NFU1 to holo-NFU1.

The relatively tight interaction between ISCU and NFU1 (*K*_d_ = 1.1 ± 0.2 μM) determined here by ITC is comparable to that between ISCU and the cysteine desulfurase complex (NIA, *K*_d_ = 1.7 ± 0.4 μM) (Cai et al., 2018). Although the NMR data show that both apo-NFU1 and holo-NFU1 interact with ISCU (Fig. 1B,C), the SEC data show that the ISCU:apo-NFU elutes as a complex (Fig. 1D), whereas ISCU:holo-NFU1 does not (Fig. 5B).

We propose that the mechanism leading to holo-NFU1 starts with formation of a 1:1 NFU1:ISCU[4Fe-4S] complex. We entertain two possible pathways for the subsequent steps leading to formation of a cluster-linked NFU1 dimer. In the first, the –SH groups of the two cysteine residues in the CTD displace two of the ISCU ligands to the [4Fe4S] cluster; this is followed by a second apo-NFU1 molecule attacking this complex with its two –SH groups displacing the other ISCU cluster ligands. In the alternative pathway, a second NFU1 molecule interacts with the initial 1:1 NFU1:ISCU[4Fe-4S] complex with its two CTD –SH groups displacing ISCU ligands to the Fe-S cluster; this is followed by displacement of the other ISCU ligands by the –SH groups of the first NFU1 molecule. Three cluster-linked dimers then associate to form the trimer of dimers representative of holo-NFU1 (Cai et al., 2016). Freed ISCU becomes available as a scaffold for subsequent cluster assembly.

The lack of transfer of a cluster from ISCU[2Fe-2S], is consistent with the evidence that NFU1 is strictly a [4Fe-4S] Fe-S protein (Melber et al., 2016; Tong et al., 2003; Cai et al., 2016). Although the NMR results presented here show that ISCU[2Fe-2S] and apo-NFU1 interact weakly, cluster transfer is not observed.

Our *in vitro* study shows that cluster transfer occurs from ISCU[4Fe-4S] to apo-NFU1 in the absence of a chaperone or co-chaperone. Interestingly, the recent study by McCarthy and Booker shows that HscA/HscB does not increase the cluster transfer rate from *E. coli* IscU [4Fe-4S] or NfuA[4Fe-4S] to LipA (McCarthy and Booker, 2017).

The present results suggest additional lines of research. It will be of interest to determine whether ISCU[4Fe-4S] binds to HSC20. The Cterminal region of HSC20, which contains residues L162 and M166, has been shown to be critical for binding to ISCU (Ciesielski et al., 2012), and this same region of HSC20 serves as a recognition site for LYR (-Leu-Tyr-Arg-) sequences on apo-Fe-S proteins that target the cluster delivery system to sites where it is needed to carry out Fe-S protein maturation (Maio and Rouault, 2015; Maio et al., 2014). Many of these target sites are on proteins that bind [4Fe-4S] clusters. This raises the question whether an ISCU[4Fe-4S]:HSC20 complex would be sufficient to deliver clusters to such proteins in the absence of a chaperone. It also would be of interest to determine whether a [4Fe-4S] cluster bound to ISCU is more labile than a [2Fe-2S] cluster, for example, as the result of steric effects.

The alternative pathway of NFU1[4Fe-4S] assembly presented here goes counter to some contemporary thinking about ISCU[4Fe-4S]. Although numerous studies have shown that ISCU can assemble both [2Fe-2S] and [4Fe-4S] clusters (Unciuleac et al., 2007; Chandramouli et al., 2007; Agar et al., 2000; Colin et al., 2013; Cai et al., 2013; Agar et al., 2000; Smith et al., 2005); recent studies have suggested that ISCU [4Fe-4S] may not be a physiologically relevant species (Fox et al., 2015; Webert et al., 2014). We show that in the presence of DTT, the [2Fe-2S] on ISCU can assemble [4Fe-4S] cluster, however, DTT is not a physiological reducing agent. It remains an open question whether there is a physiological reducing agent that is capable of achieving that. In addition, a recent study showed that [2Fe-2S]-GLRX5-BOLA3 complex is able to assemble a [4Fe-4S] cluster on NFU1 in presence of reducing agents such as DTT (Nasta et al., 2019). Future *in vivo* experiments are needed to test the physiological relevance of this alternative pathway.

## 4. Materials and methods

### 4.1. Proteins and buffers

The HNT buffer used in this study consisted of 50 mM HEPES pH 7.5, 150 mM NaCl, and 5 mM TCEP (HNT buffer), and the HN buffer consisted of 50 mM HEPES pH 7.8, 150 mM NaCl. All the buffers were clarified by passage through a 0.2 μm filter (Millipore; Billerica, MA) prior to use. For anaerobic experiments, the buffer was thoroughly degassed and equilibrated for at least 12 h in an anaerobic chamber (Coy Laboratory; Farmingdale, NY) filled with 95% N_2_ gas and 5% H_2_ gas. The O_2_ level in the anaerobic chamber was kept below 1 ppm.

We used published procedures to prepare ISCU and ISCU(D39V) (Cai et al., 2013). Apo- and holo-NFU1 were prepared as described earlier (Cai et al., 2016). Non-isotope-labeled proteins were expressed in auto-induction media (Studier, 2005). ^15^N-labeled proteins were expressed in M9 minimal media (Jansson et al., 1996) containing ^15^N-ammonium chloride as the sole nitrogen source.

### 4.2. Fe-S cluster reconstitution on ISCU

ISCU[2Fe-2S] was prepared by a protocol described earlier (Cai et al., 2017). Briefly, inside an anaerobic chamber anaerobic, HN buffer was prepared containing 0.2 mM ISCU, 2 μM NIA, 0.2 mM reduced FDX2, and 0.4 mM Fe(NH_4_)_2_(SO_4_)_2_. 0.4 mM L-cysteine was added to initiate to Fe-S cluster assembly reaction. Under these conditions, only ISCU[2Fe-2S] is generated (Webert et al., 2014). After incubation for 4 h, the reaction mixture was desalted on a Zeba Spin desalting column (Thermo Fisher Scientific) and then purified on a HiTrap Q HP anion exchange column (GE Healthcare). A salt gradient from 0 to 500 mM NaCl was used to elute the proteins. Apo-ISCU (pI = 9.21) does not bind to the anion exchange column at pH 7.5. Both ISCU[2Fe-2S] and FDX2 bind to the anion exchange column, but are eluted differentially by the salt gradient. The concentration of [2Fe-2S] cluster was estimated from the extinction coefficient ε_410nm_ = 8000 M^−1^cm^−1^ for [2Fe-2S]^2+^.

ISCU[4Fe-4S] was prepared using a protocol as described earlier with modifications (Cai et al., 2013). Briefly, 0.1 mM ISCU, 2 μM NIA, 0.5 mM Fe(NH_4_)_2_(SO_4_)_2_ and 5 mM DTT was mixed together in anaerobic HN buffer inside an anaerobic chamber. The Fe-S reconstitute reaction was initiated by adding 0.5 mM L-cysteine into the reconstitution mix. Under these conditions, both ISCU[2Fe-2S] and ISCU [4Fe-4S] are generated. After 5 h of incubation, the excess reducing agent DTT converted the ISCU[2Fe-2S] to ISCU[4Fe-4S]. The reconstitution product was confirmed by UV–vis spectroscopy on a UV-1700 spectrophotometer (Shimadzu; Kyoto,Japan), which showed only one broad peak at 410 nm. The ISCU[4Fe-4S] concentration was estimated from the extinction coefficient ε_410_ nm = 15,000 M^−1^cm^−1^ for [4Fe4S]^2+^. The reaction mix was subsequently dialyzed thoroughly against anaerobic HN buffer to remove excess Fe(NH_4_)_2_(SO_4_)_2_, DTT, and L-cysteine.

### 4.3. NMR spectroscopy

The HNT buffer used for NMR samples contained 8% D_2_O for the frequency lock. All NMR spectra were collected at the National Magnetic Resonance Facility at Madison (NMRFAM) on 600 or 750 MHz (^1^H) Bruker BioSpin (Billerica, MA) NMR spectrometers equipped with a z-gradient cryogenic probe. All sample temperatures were regulated at 25 °C. NMRPipe software (Delaglio et al., 1995) was used to process the raw NMR data, and NMRFAM-SPARKY (Lee et al., 2015) software was utilized to visualize and analyze the processed NMR data. 0.3 mM [U-^15^N]-apo-NFU1 samples in HNT buffer were prepared in the anaerobic chamber (O_2_ < 1 ppm) to prevent cysteine oxidation. The samples were then transferred in the anaerobic chamber to NMR tubes (Wilmad-Labglass) equipped with gas-tight seals. To monitor the effects of added unlabeled ISCU on [U-^15^N]-NFU1, solutions of the two proteins in HNT buffer were mixed to achieve equimolar concentrations, and 2D ^1^H,^15^N TROSY-HSQC spectra were acquired. An analogous approach was used in preparing the samples used to determine the effects of added apo- or holo-NFU1 on [U-^15^N]-ISCU.

The NMR sample used to investigate whether ISCU[4Fe-4S] transfers its cluster to apo-NFU1 was prepared as follows. In the anaerobic chamber, ISCU[4Fe-4S] produced from the Fe-S cluster assembly reaction was dialyzed to remove free iron and sulfide. Then solutions of [U-^15^N]-apo-NFU1 and unlabeled ISCU[4Fe-4S] in HNT buffer were mixed and incubated in the anaerobic chamber for 30 min; after incubation, the sample was transferred to an NMR tube with a gas-tight seal, and a 2D ^1^H,^15^N TROSY-HSQC spectrum was recorded. The NMR sample used to investigate whether ISCU[2Fe-2S] transfers its cluster to apo-NFU1 was prepared as follows. In the anaerobic chamber, solutions of [U-^15^N]-ISCU[2Fe-2S] and NFU1 in HNT buffer were mixed to achieve a 2:1 M equivalence. The mixture was incubated in the aerobic chamber for 10 h and then transferred to an NMR tube with a gas-tight seal, and a 2D ^1^H,^15^N TROSY-HSQC spectrum was recorded. To investigate the effect of DTT on the cluster transfer, 5 mM DTT was added to the above mix and incubated for 4 h in the anaerobic chamber; the sample was subsequently transferred to a gas-tight NMR tube, and a 2D ^1^H,^15^N TROSY-HSQC spectrum was recorded.

### 4.4. Isothermal titration calorimetry

A Nano ITC system (TA Instruments) was used to investigate the interactions between ISCU and apo-NFU1. Proteins were dialyzed overnight in the HNT buffer and degassed thoroughly before the ITC experiment. The ITC experiment was conducted at 25 °C. The syringe contained 0.8 mM ISCU, and the sample cell (169 μL) contained 0.03 mM NFU1. 20 2.5 μL aliquots of the sample in the syringe were injected into the solution in the sample cell, and the heat generated was measured. The ITC data processing and fitting were conducted using NanoAnalyse Software (TA Instruments).

### 4.5. Size exclusion chromatography

To investigate the interaction between NFU1 and ISCU, size exclusion chromatography (SEC) experiments were carried out on a HiLoad Superdex 75 PG gel filtration column (GE Healthcare). Samples of NFU1, ISCU or a mixture of ISCU:NFU1 at 3:1 molar ratio were injected separately onto the column and eluted by HNT buffer. The flow rate was kept at 1 mL/min, and an automatic fraction collector (GE Healthcare) was used to collect the eluate. To determine the protein identity and purity, a 10 μL aliquot from each fraction was subjected to SDS-PAGE analysis.

To study whether ISCU[4Fe-4S] transfers its cluster to apo-NFU1, a 1:1 mixture of [U-^15^N]-apo-NFU1 and ISCU[4Fe-4S] was incubated in the anaerobic chamber for 30 min before being injected onto a HiLoad Superdex 200 PG gel filtration column (GE Healthcare). The proteins were eluted by HNT buffer at a flow rate of 1 mL/min, and fractions were collected. To determine the protein identity and purity, a 10 μL aliquot from each fraction was subjected to SDS-PAGE analysis.

### 4.6. SAXS data acquisition and analysis

The apo-NFU1:ISCU complex used for SAXS analysis was isolated by SEC, and the fraction containing the complex was dialyzed extensively against HNT buffer with three buffer changes. The solution was clarified by passage through a 0.2 μm filter. SAXS data were collected from apo-NFU1:ISCU samples prepared at three different concentrations ranging from 2 to 8 mg/mL. No significant interparticle interactions were observed at any of these concentrations. SAXS experiments were carried out on a Nanostar benchtop SAXS system (Bruker AXS) at NMRFAM, which was equipped with a rotating anode (Cu) Turbo X-ray Source and a VÅNTEC-2000 (2048 × 2048 pixel) detector. The sampleto-detector distance was set at ~1 m, allowing for the detection range: 0.012 > *q* > 0.250 Å^−1^. 40 μL protein and buffer samples were loaded separately into a capillary cell with 1 mm diameter, and scattering data were collected over a 3 h period with frames recorded every hour. Each frame was compared to check for radiation damage, and none was detected over the course of the experiments. The SAXS data sets were then averaged and converted to 1D scattering profiles for further analysis.

The ATSAS (Petoukhov et al., 2012) software suite was used to process the SAXS data. The radius of gyration (*R*g) for each protein or protein complex was determined by using the Guinier approximation in the q range (qmax·*R*_g_) < 1.3. Pairwise distance distribution functions (*P*r) were obtained using the software GNOM (Svergun, 1992). The output from GNOM was then used in conjunction with DAMMIF (Franke and Svergun, 2009) to generate 20 independent *ab initio* dummy atom models to assess the molecular shape of each sample. Most of the models exhibited excellent agreement with experimental data and had a normalized spatial discrepancy (NSD) < 1. We used the software HADDOCK (van Zundert et al., 2016; de Vries et al., 2010) to carry out rigid body modeling, and CRYSOL (Svergun et al., 1995) to compare the models resulting from the rigid body modeling to experimental data. *V*c approach was used for the molecular mass calculation from the SAXS data (Rambo and Tainer, 2013). Supcomb software (Kozin and Svergun, 2001) was used to superimpose protein structures on to the SAXS *ab initio* dummy atom models.

### 4.7. HADDOCK modeling

The HADDOCK2.2 webserver (van Zundert et al., 2016; de Vries et al., 2010) was used to generate the structural model of NFU1-ISCU complex. The ISCU structure was taken from the NMR structure of Zn^2+^ bound mouse ISCU, which shares 98% sequence identity to human ISCU (pdb: 1wfz). The full-length NFU1 structure was generated by fitting the NMR structures of the CTD (pdb: 2m5o) and NTD (pdb: 2ltm) of NFU1 into the previously reported SAXS density of full-length NFU1 (Cai et al., 2016). Chemical shift perturbations observed upon complex formation were used to define ambiguous interaction constraints (AIRs). Active residues were defined as those with severe peak broadening. Passive AIRs were defined automatically. A total of 5,000 rigid-body docking trials were carried out using the standard HADDOCK protocol. The 200 lowest-energy solutions were used for subsequent semiflexible simulated annealing and water refinement. The HADDOCK software sorted the 192 structures into 4 clusters, which represented 96.0% of the water-refined models generated. The statistics of the top three clusters are reported in Table 2.

## Supplementary Material

2

## Figures and Tables

**Table 1:** Summary of SAXS data on the NFU1-ISCU complex.

Data-collection parameters	
Instrument	Bruker Nanostar
Beam geometry	2 pinholes (500 μm)
Wavelength (Å)	1.5418
*q* range (Å^−1^)	0.012 – 0.250
Exposure time (h)	3
Concentration range (mg ml^−1^)	2 – 8
Temperature (K)	298
I(0) [from *P*(r)]	1212 ± 10
R_g_ (Å)[from *P*(r)]	35.9 ± 0.5
I(0) (from Guinier)	1242 ± 28
*R*_g_ (Å) (from Guinier)	35.8 ± 1.0
*D*_max_ (Å)	97.6 ± 5
Dry volume calculated from sequence (10^3^ Å^3^)	50.4
Porod volume estimate (10^3^ Å^3^)	51.2
**Molecular-mass determination**	
Molecular mass [*V*_c_] (kDa)	42.8 ± 3
Calculated molecular mass from sequence (kDa)	41.7
**Ab initio Modeling**	
Number of models	20
NSD	0.551 ± 0.014
χ^2^	1.36
**Software employed**	
Primary data reduction	SAXS (Bruker)
Data processing	PRIMUS
*Ab initio* analysis	DAMMIF
Validation and averaging	DAMAVER
Superimposition	SUPCOMB
Computation of model intensities	CRYSOL
Three-dimensional graphics representations	PYMOL

**Table 2:** Statistics of the HADDOCK docking of NFU1 and ISCU.

	Cluster 1	Cluster 2	Cluster 3
HADDOCK score	−114.3 ± 4.6	−102.2 ± 7.2	−100.8 ± 5.0
Cluster size	111	45	25
RMSD^a^	1.3 ± 0.7	3.7 ± 0.2	3.9 ± 0.5
Van der Waals energy	−55.0 ± 2.8	−60.5 ± 6.5	−46.6 ± 2.3
Electrostatic energy	−318.8 ± 27.2	−216.7 ± 23.9	−343.2 ± 15.9
Desolvation energy	−12.3 ± 3.1	−8.4 ± 6.5	1.2 ± 6.4
Restraints violation energy	167.6 ± 45.44	99.9 ± 22.88	133.0 ± 48.32
Buried Surface Area	1630.6 ± 83.9	1711.6 ± 56.5	1558.5 ± 59.9
Z-Score^b^	−1.1	−0.3	−0.2

a: RMSD from the overall lowest-energy structure.

b: Z-score indicates how many standard deviations from the average this cluster is located in terms of score.
