# Supplementary material for: ISCU interacts with NFU1, and ISCU[4Fe-4S] transfers its Fe-S cluster to NFU1 leading to the production of holo-NFU1
Source: J Struct Biol. Author manuscript; Available in PMC 2021 May 1. (PMC7261492; doi:10.1016/j.jsb.2020.107491)
Supplement: 2 [file NIHMS1575985-supplement-2.docx]

Supplementary Materials


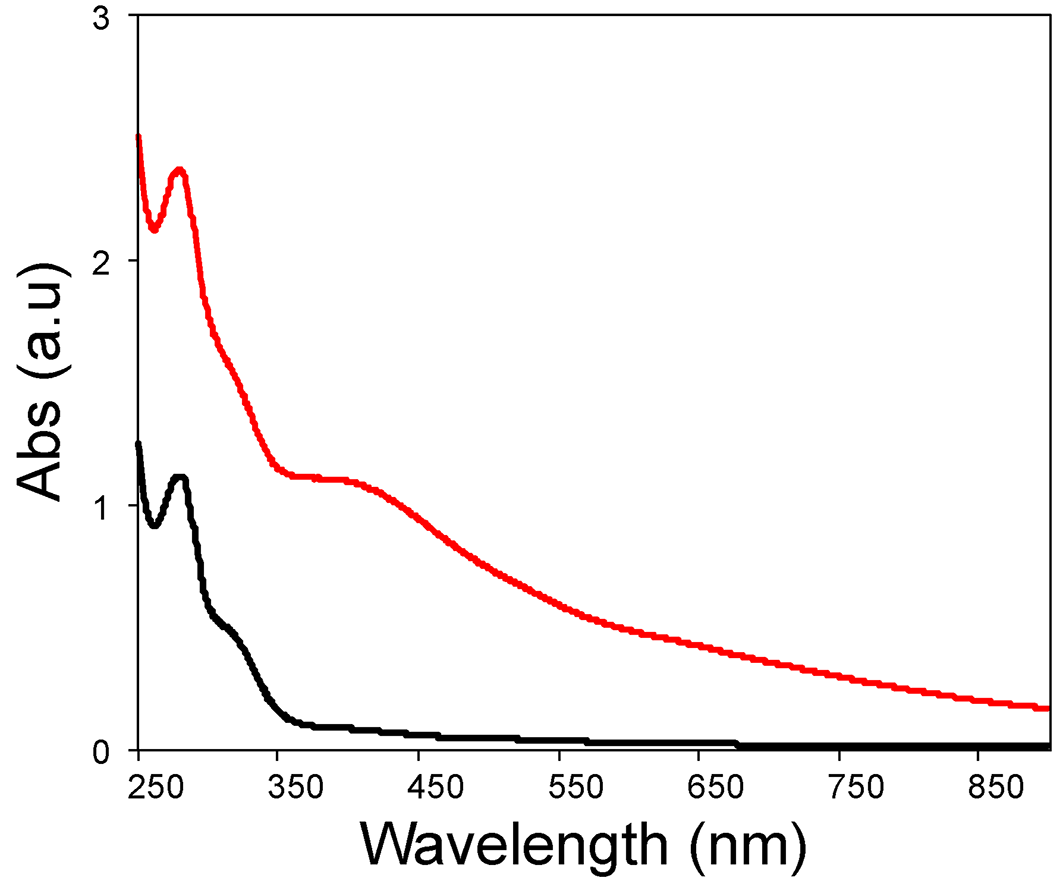


**Supplementary Figure 1.** UV-vis spectra of Fe-S cluster reconstitution reaction mix (0.1 mM ISCU, 2 µM NIA, 0.5 mM Fe(NH_4_)_2_(SO_4_)_2_, 5mM DTT and 0.5 mM L-cysteine) before (black line) and after (red line) *in vitro* Fe-S cluster assembly assay. The single broad peak at ~410 nm indicates the presence of [4Fe-4S] cluster.


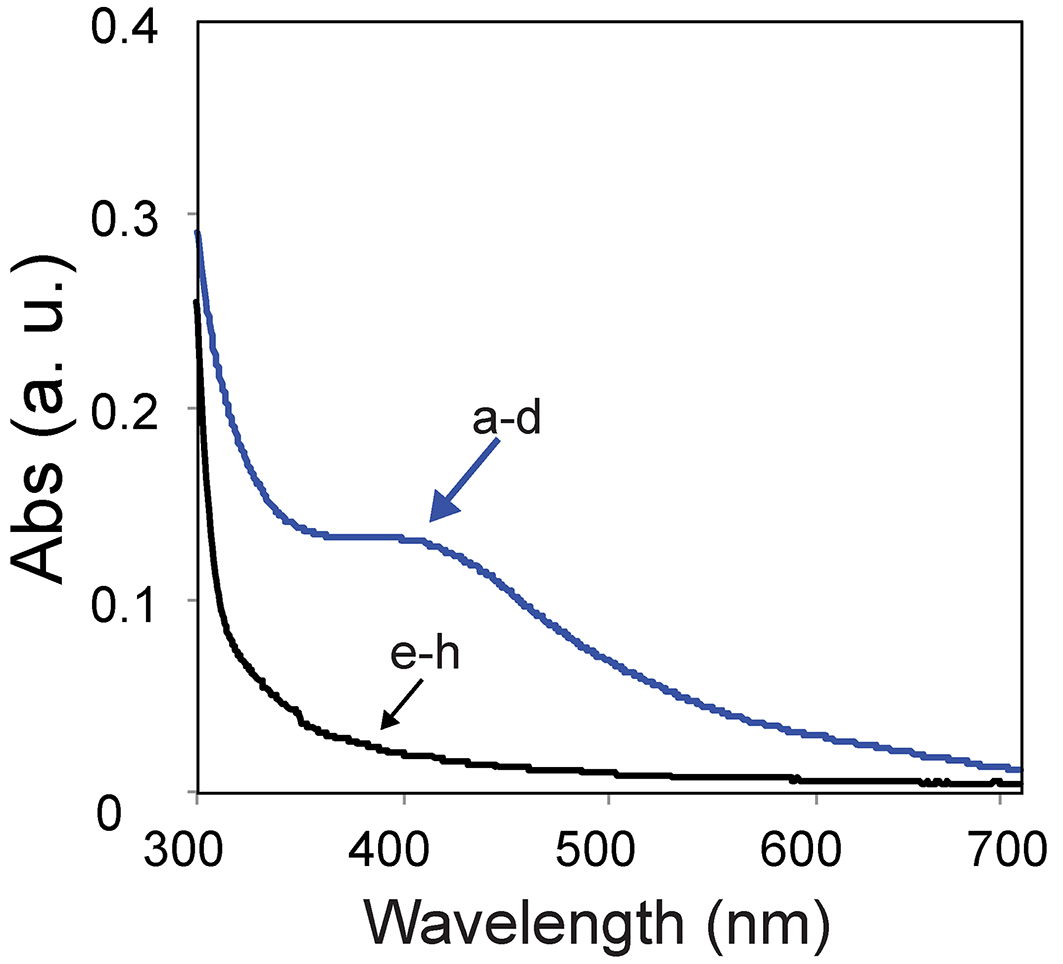


**Supplementary Figure 2.** (related to Figure 5B) blue line, UV-vis spectra of elution fractions a-d in Fig 5B pooled together; black line, UV-vis spectra of elution fractions e-h in Fig 5B pooled together.
